# Blood–Brain Barrier and Neurovascular Unit In Vitro Models for Studying Mitochondria-Driven Molecular Mechanisms of Neurodegeneration

**DOI:** 10.3390/ijms22094661

**Published:** 2021-04-28

**Authors:** Alla B. Salmina, Ekaterina V. Kharitonova, Yana V. Gorina, Elena A. Teplyashina, Natalia A. Malinovskaya, Elena D. Khilazheva, Angelina I. Mosyagina, Andrey V. Morgun, Anton N. Shuvaev, Vladimir V. Salmin, Olga L. Lopatina, Yulia K. Komleva

**Affiliations:** 1 Research Institute of Molecular Medicine and Pathobiochemistry, Prof. V.F. Voino-Yasenetsky Krasnoyarsk State Medical University, 660022 Krasnoyarsk, Russia; ekaterinav1201@gmail.com (E.V.K.); yana_20@bk.ru (Y.V.G.); elenateplyashina@mail.ru (E.A.T.); malinovskaya-na@mail.ru (N.A.M.); elena.hilazheva@mail.ru (E.D.K.); angelina.mosiagina@gmail.com (A.I.M.); 441682@mail.ru (A.V.M.); shuvaevanton@hotmail.com (A.N.S.); vsalmin@gmail.com (V.V.S.); ol.lopatina@gmail.com (O.L.L.); yuliakomleva@mail.ru (Y.K.K.); 2 Research Center of Neurology, 125367 Moscow, Russia

**Keywords:** neurodegeneration, in vitro blood–brain barrier model, in vitro neurovascular unit model, mitochondria, Alzheimer’s disease

## Abstract

Pathophysiology of chronic neurodegeneration is mainly based on complex mechanisms related to aberrant signal transduction, excitation/inhibition imbalance, excitotoxicity, synaptic dysfunction, oxidative stress, proteotoxicity and protein misfolding, local insulin resistance and metabolic dysfunction, excessive cell death, development of glia-supported neuroinflammation, and failure of neurogenesis. These mechanisms tightly associate with dramatic alterations in the structure and activity of the neurovascular unit (NVU) and the blood–brain barrier (BBB). NVU is an ensemble of brain cells (brain microvessel endothelial cells (BMECs), astrocytes, pericytes, neurons, and microglia) serving for the adjustment of cell-to-cell interactions, metabolic coupling, local microcirculation, and neuronal excitability to the actual needs of the brain. The part of the NVU known as a BBB controls selective access of endogenous and exogenous molecules to the brain tissue and efflux of metabolites to the blood, thereby providing maintenance of brain chemical homeostasis critical for efficient signal transduction and brain plasticity. In Alzheimer’s disease, mitochondria are the target organelles for amyloid-induced neurodegeneration and alterations in NVU metabolic coupling or BBB breakdown. In this review we discuss understandings on mitochondria-driven NVU and BBB dysfunction, and how it might be studied in current and prospective NVU/BBB in vitro models for finding new approaches for the efficient pharmacotherapy of Alzheimer’s disease.

## 1. Introduction

Neurovascular unit (NVU) is a complex element consisting of brain microvessel endothelial cells (BMECs), pericytes, perivascular astrocytes, neurons, oligodendrocytes, and microglia, which are in close cooperation being responsive to changes in local microcirculation and metabolism. Actually, the functional activity of cells within the NVU dictates how brain microvessels could adapt the blood flow to the local needs. Particularly, active brain regions require more nutrients and oxygen; therefore, high extracellular concentrations of potassium and lactate would stimulate elevated blood flow (so-called gliovascular control of microcirculation), whereas stem and progenitor cells within neurogenic niches require the maintenance of a local microenvironment for the adequate response to pro-neurogenic stimuli coming from activated or injured brain regions [1,2]. Thus, NVU links the activity of neurons and microcirculation as well as the metabolic needs of specific cell populations and local microenvironments. Therefore, the blood–brain barrier (BBB) controlling passage of nutrients, metabolites, and regulatory molecules into and from the brain parenchyma is recognized as an important part of the NVU. BBB consists of brain microvessels, endothelial cells (BMECs), pericytes, astrocytes, and basement membranes serving as a matrix for BBB cells. All the cells within the BBB are in tight contact and cooperation to provide selective and controlled transport of molecules (metabolites, cytokines, xenobiotics, etc.) and cells (blood immune cells, bacteria) through the barrier. Actually, the functional activity of BBB cannot be viewed without NVU because activation of neurons always corresponds to significant changes in local microcirculation and BBB permeability.

The functional activity of the BBB, which is important for the maintenance of many key functions of the central nervous system, becomes an obstacle in the use of many pharmaceutical agents: most of the compounds used as drugs and their carriers do not reach their molecular targets in brain cells due to limited and selective BBB permeability. This results in a serious problem for the treatment of a wide range of mental and neurological diseases. On the other hand, being abnormally increased, uncontrolled BBB permeability is a characteristic of brain disorders (chronic neurodegeneration, stroke, migraine, epilepsy, brain tumors, brain trauma, perinatal brain injury, neuroinfections, etc.), contributing to the development of serious complications (e.g., brain edema, microbleedings) and neurological deficits [3,4]. Thus, the study of cellular and molecular mechanisms of BBB and NVU damage and recovery is a large and significant part of modern basic and clinical neuroscience.

It is commonly accepted that progression of chronic neurodegeneration is based on aberrant signal transduction, excitation/inhibition imbalance, excitotoxicity, synaptic dysfunction, oxidative stress, proteotoxicity and protein misfolding, local metabolic dysfunction, excessive cell death, development of glia-supported neuroinflammation, and failure of neurogenesis [5,6]. All the above-mentioned mechanisms directly relate to dramatic changes in the structure and activity of the NVU (aberrant neuron-astrocyte metabolic coupling, deregulated microcirculation, etc.) and BBB (excessive neoangigenesis, BBB breakdown, etc.) Dysfunction of NVU and BBB contributes to the progression of chronic neurodegeneration, and should be considered as a target mechanism for reducing brain damage and corresponding neurological deficits.

In the last decade, mitochondrial and metabolic alterations in brain cells appear in the focus of researchers. NVU/BBB cells of neuronal, glial, and endothelial origins have quite different and complicated mechanisms of energy production for supporting their functional activity, proliferation, differentiation, and response to the action of external stimuli. That is why analysis of mitochondria-driven processes could shed a light on the key mechanisms of brain plasticity in (patho)physiological conditions. Particularly, in Alzheimer’s type neurodegeneration, mitochondria serve as a platform for the action of misfolded proteins, initiation and progression of cell death and the development of Ca2+ imbalance, but at the same time, they should be considered as promising therapeutic targets [7]. However, focus on the assessment of mitochondrial alterations in neurons should be extended to an analysis of mitochondrial and metabolic changes induced in non-neuronal cells in Alzheimer’s disease.

## 2. Mitochondrial Dysfunction and NVU/BBB Impairment in Alzheimer’s Type Neurodegeneration

Alzheimer’s type neurodegeneration has an incompletely deciphered pathogenesis consisting of numerous complementary mechanisms such as amyloid beta (Aβ) cytotoxicity, impaired neuronal excitability and neuron–glia interactions, neuroinflammation, cerebral amyloid angiopathy, local insulin resistance, impaired neurogenesis, development of phenomenon of hypervascularization and impaired permeability of BBB [8,9,10,11,12]. It is suggested that many mechanisms of aberrant brain plasticity in Alzheimer’s disease could be linked to mitochondrial dysfunction [7].

In mammalian cells, mitochondria are not usually present as separate organelles, but form a tubular network of interconnected structures that are constantly changing due to fusion or separation (fission), biogenesis or mitophagy [13]. Neuronal and non-neuronal NVU cells vary in their ability to coordinate all these processes because they have a different proliferation status (i.e., postmitotic neurons vs proliferating angiogenic endothelial cells), energy requirements (i.e., quiescent neural stem cells vs activated progenitor cells), intracellular location of mitochondria (i.e., synaptic accumulation in neurons vs distribution in the cell body in endothelial cells), and support from other energy-producing processes (i.e., excessive glycolysis in astrocytes vs low glycolytic activity in neurons) [7,14]. In sum, all these characteristics, as well as the developmental stage and brain region, should be taken into consideration while analysis of brain tissue aims to identify cell-specific mitochondrial activity.

The main factors regulating mitochondrial dynamics have been identified, in particular, intracellular proteins like dynamin-related protein Drp1 that controls mitochondrial fission/fragmentation, mitofusins MFN1, MFN2 that controls mitochondrial fusion [14], peroxysome proliferator-activating receptor PGC-1α, and nuclear respiratory factors NRF1 and NRF2 that control mitochondrial biogenesis [15]. In general, mitochondrial fusion and fission adapt mitochondrial morphology to the current metabolic needs of a cell: fragmented mitochondria are usually observed in resting cells or when autophagy is required for cell clearance [16]. In pathological conditions, the prevalence of mitochondrial fragmentation or fusion may signal on the metabolic activity of cells. For instance, aberrant mitochondrial dynamics in affected cells is always seen in neuroinflammation or in hypometabolic conditions in the brain caused by insulin resistance due to impaired implementation of the mitochondrial stress response controlled by the effects of insulin [17,18] that are the essential components of Alzheimer’s disease pathogenesis.

### 2.1. Aberrant Mitochondrial Function and Dynamics in Neurons

It is known that the activity of neurons in the brain is largely determined by the preservation of the functions of mitochondria, whose accumulation in the perisynaptic region ensures the effective secretion and reception of neurotransmitters, whereas their presence in the cell soma supports the synthesis of proteins for subsequent axonal transport [14]. Direct action of Aβ on neuronal mitochondria may trigger mitochondrial dysfunction, excessive fragmentation and an abnormal mitochondrial network, thereby leading to the loss of the metabolic and integrative function of mitochondria, synaptic failure and aberrant neuroplasticity. Failure of mitostasis in neurons as non-dividing cells might be extremely important for the progression of neurodegeneration [14].

Considering that mitochondria control many cellular events like the final stages of catabolism leading to production of ATP, cell viability and its sensitivity to the action of factors that induce apoptosis, necrosis and autophagy, maintaining ion homeostasis, signal transduction, any mitochondrial damage in Alzheimer’s disease has significant consequences for neurons. Therefore, it is not surprising that damage to the mitochondria of neurons in Alzheimer’s type neurodegeneration is considered as an integral mechanism of the disease progression: toxic action of Aβ, Ca2+ imbalance and overproduction of reactive oxygen species have been reported as the main causes and outcomes of mitochondrial dysfunction [19,20] being closely associated with energy deficit in neurons due to glucose hypometabolism and development of local insulin resistance [21]. Aβ-induced deregulation of local Ca2+ signaling due to mitochondrial dysfunction results in ROS overproduction in mitochondria, development of oxidative stress, and progression of cell death (apoptosis or autophagy) [22,23].

**Figure 1:**
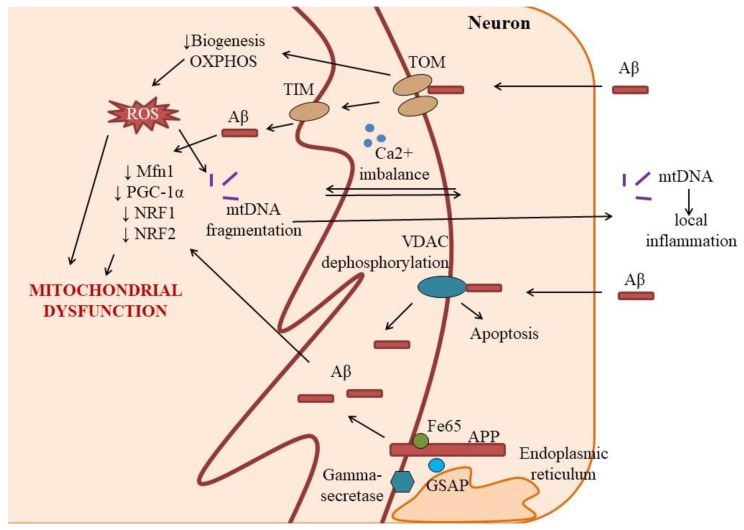
**The role of mitochondrial DNA damage to the local inflammation, glial activation, and secondary tissue destruction.** mtDNA—mitochondrial DNA; OXPHOS—oxidative phosphorylation; Mfn—mitofusins; PGC—1α—peroxysome proliferator-activating receptor; NRF1 and NRF2—nuclear respiratory factors; Aβ—amylod beta; TOM—translocase of the outer membrane; VDAC—voltage-dependent anion channel; GSAP—gamma-secretase activating protein; APP—amyloid precursor protein.

Several authors report the presence of mitochondrial DNA (mtDNA) damage and loss in Alzheimer’s disease, or increase in risk of Alzheimer’s disease development by inherited mtDNA variants [24,25], but the impact of this mechanism on the pathogenesis of Alzheimer’s disease remains unclear. However, the release of short fragments of mtDNA serving as mitochondrial damage-associated molecular pattern (DAMPs) in the extracellular space may trigger local inflammation, glial activation, and secondary tissue destruction (Figure 1) [26].

There is accumulating evidence that Aβ can directly disrupt the activity of neuronal mitochondria by binding to mitochondrial proteins, resulting in the disruption of their conformation and functional activity. It was shown in vitro and in vivo that Aβ can be transported into mitochondria via the translocase of the outer membrane (TOM) complex for further accumulation in the mitochondrial matrix [27]. TOM complex is known as a site for binding the amyloid precursor protein (APP) in mitochondria, leading to impaired protein transport and suppression of mitochondrial ATP synthesis [28]. TOM machinery with a TIM complex (translocase of inner membrane) and heat shock proteins (hsp70) take part in the mitochondrial biogenesis, providing protein import into the matrix [29]. Thus, it is very reasonable to assume that binding of Aβ to TOM would result in suppression of biogenesis and perturbations of oxidative phosphorylation (OXPHOS) complex in neurons [30].

In some cases, mitochondrial proteins may control the entry of Aβ into the cell: voltage-dependent anion channels (VDACs) expressed in the mitochondria outer membrane within the mitochondrial permeability transition pore (MPTP) interacts with Aβ, thereby leading to its transport inside the cell and induction of apoptosis [31]. In physiological conditions, VDAC is phosphorylated by glycogen synthase kinase-3 (GSK3 kinase) [32], but Aβ promotes dephosphorylation of VDAC required for apoptosis progression coupled to matrix swelling, membrane depolarization and cytochrome c release [33].

Gamma-secretase activating protein (GSAP), which stimulates proteolysis of APP towards Aβ is enriched in mitochondria-associated membrane of the endoplasmic reticulum and interacts with APP-Fe65 complex to control APP proteolysis. It is involved in the pathogenesis of mitochondrial dysfunction and cognitive decline in Alzheimer’s disease [34]. Vice versa, mitochondrial dysfunction leads to aberrant processing of APP: inhibition of glycolysis and mitochondrial respiration as a model of metabolic stress impairs APP proteolysis and increases the probability of amyloidogenesis [35].

Disturbance of mitochondrial dynamics (fusion and fission of mitochondria, mitochondrial biogenesis) is a characteristic of mitochondria in Alzheimer’s disease, which indicates a change in the regulation of processes associated with ensuring a stable population of mitochondria in neurons—mitostasis [14,36]. An important feature of the mitochondrial pool in neurons is that most of the mitochondria are distanced from the cell soma, being present in neurites where mitochondria support synaptic transmission. According to some reports, a neuron actively involved in synaptic activity may contain up to 2 million mitochondria [14]; so, the importance of the preserved mitochondrial pool for synaptic plasticity can hardly be overestimated. However, in Alzheimer’s disease, mitochondrial mass is reduced due to impaired mitochondrial biogenesis and low expression of PGC-1α, NRF1, and NRF2 as was demonstrated in human hippocampal neurons as well as in cells overexpressing APP causing familial Alzheimer’s disease [37]. These data have been later extended to the Aβ toxicity in vitro model where Aβ produced a reduction in Mfn1 and PGC-1α levels, dramatic changes in the mitochondrial network [38].

**Figure 2:**
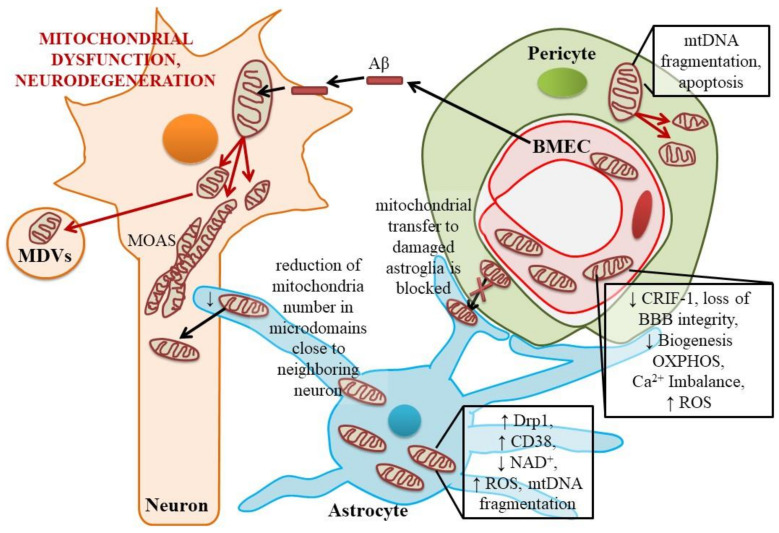
Impaired mitochondrial function in Alzheimer’s disease within neurovascular unit. MDVs—mitochondria-derived vesicles; MOAS—mitochondria-on-a-string; mtDNA—mitochondrial DNA; Aβ—amylod beta; BMEC—brain microvessel endothelial cells; Drp1—dynamin-related protein 1; CD38—cluster of differentiation 38; ROS—reactive oxygen species; CRIF-1—CR6-interacting factor 1; BBB—blood–brain barrier; OXPHOS—oxidative phosphorylation.

Interestingly, a new phenotype of neuronal mitochondria in Alzheimer’s disease was identified as MOAS (mitochondria-on-a-string): cells are characterized by the presence of elongated, interconnected mitochondria, and the same MOAS organelles are found during cell aging and hypoxic damage (Figure 2) [39]. Presumably, development of MOAS reflects the common mechanism of protection against mitophagy to preserve the remaining mitochondria in the cell via blocking the fission machinery [39]. Some authors attribute the molecular mechanisms of mitochondrial damage to the fact that the “quality control” of mitochondria is disturbed in cells, leading to excessive mitophagy/autophagy [40]. These events are closely related to the development of oxidative stress, accumulation of misfolded proteins in the cell, and calcium mitochondrial imbalance. When damaged, mitochondria can initiate the activation of intramitochondrial proteases, mitophagy, and may release into cytosol several proapoptotic proteins. Another way is to incorporate mitochondria or their fragments into membrane-derived microvesicles or exosomes that appear in the extracellular space when cells are damaged or overactivated. Some immune cells can prevent packaging of oxidatively damaged mitochondrial proteins into extracellular vesicles (EVs) to reduce the pro-inflammatory potential of EVs, and instead target these proteins in a form of mitochondria-derived vesicles (MDVs) for intracellular lysosomal degradation [41]. However, this mechanism was not examined in neuronal cells yet. Moreover, Aβ-mediated disruption of nutrient-induced mitochondrial activity as a communication between neuronal lysosomes and mitochondria [42] has been proposed as a mechanism of neuronal damage in Alzheimer’s disease. Restoration of mitophagy and inhibition of mitochondrial fission-promoting factor Drp1, whose interaction with Aβ results in defective mitophagy, might be beneficial for preventive neuronal death and cognitive deficits in Alzheimer’s type neurodegeneration [43]. However, some studies [44] have revealed that mitophagy is inhibited in neurons in Alzheimer’s disease, and stimulation of mitophagy might have a positive effect on the cognitive functions of experimental animals. Therefore, the exact role of aberrant mitophagy in neuronal damage in Alzheimer’s disease needs further clarification.

Being not well-equipped with glycolytic machinery, neurons cannot switch from defective OXPHOS to glycolysis in the conditions of cell stress; however, aging and degenerating neurons may develop a so-called inverse Warburg effect by upregulating the activity of OXPHOS supported by pyruvate converted from astroglia-provided lactate [45]. Excessive activity of mitochondria respiratory chains would ultimately lead to ROS overproduction and progression of neurodegeneration.

### 2.2. Dysfunctional Mitochondria in Astroglial Cells

As we mentioned above, under certain conditions, the maintenance of the functional activity of damaged mitochondria in neurons can be provided by surrounding astrocytes. It is known that astrocytes, in contrast to neurons, to a greater extent provide their energy needs due to glycolytic production of ATP, maintain the functional activity of mitochondria for a long time even under unfavorable conditions, transport lactate formed in glycolysis to neurons where its conversion into pyruvate partially replenishes the needs of the tricarboxylic acid (TCA) cycle (this mechanism is known as neuron-astroglial metabolic coupling or astrocyte-neuron lactate shuttle, ANLS) [46,47]. Expression of molecules that provide the transport of lactate between astrocytes and neurons, in particular monocarboxylate transporters (MCT), determines the efficiency of the processes of memory formation and learning in the hippocampus, neuronal survival, and functional activity [48]. Stimulation of astroglial glycolysis by GLP-1 (glucagon-like peptide-1) was recently shown to be effective in supporting neuronal OXPHOS, improving cognitive abilities and suppressing oxidative stress [49]. ANLS was initially observed in glutamate-stimulated neurons [50], but some later studies revealed that activated neurons might not be so much dependent on lactate supply from astroglia since they prefer to utilize glucose to support their energetic needs [51]. In sum, there are still some controversies in the interpretation of data obtained on ANLS, but cAMP-facilitated transport of lactate from hippocampal astrocytes to neurons was very recently confirmed with optogenetic stimulation of mouse astrocytes in vivo [52].

In addition to such a “distant” action (due to the production and transport of lactate into neurons), astrocytes can directly support the mitochondrial activity in neighboring neurons. Astrocytes are equipped with mitochondria which are used for energy production as well as for maintaining intracellular Ca2+ homeostasis, particularly in cytosolic microdomains close to the contacts with neuronal cells [53]. Like in neurons, Aβ affects astroglial mitochondria, which is associated with glia activation, astrocytosis, and progression of neuroinflammation [54]. As expected, in neurodegeneration, reduction in the astroglial mitochondria number is detected in microdomains of astrocytic processes close to neighboring neurons where Ca2+ signaling is regulated by the opening of mitochondrial MPTP [55,56]. Suppression of mitochondrial activity in astrocytes causes inhibitions of glutamate uptake, presumably due to co-compartmentalization of glutamate transporter CLT-1 with the mitochondrial fraction and glycolytic domain in astrocytes [57]. As a result, glutamate is accumulated in the extracellular space due to neuronal activity being the cause of excitotoxic injury of neurons and astroglial apoptosis [58].

Neuroinflammation associated with neurodegeneration is governed by astroglial proliferation, which is supported by extensive glycolytic and mitochondrial production of ATP [59]; thus, mitochondrial dynamics in astroglial cells in Alzheimer’s type neurodegeneration might follow distinct scenarios: (i) reduction of mitochondrial activity, intensive production of ROS, activation of Drp1-dependent mitochondrial fission or mitophagy in local microdomains close to affected neurons; (ii) stimulation of mitochondrial biogenesis to restore mitochondrial network in reactive astrocytes exposed to pro-inflammatory stimuli [60]. It is interesting to note that induction of mtDNA loss in astrocytes results in their activation and progression of neuroinflammation in vivo [61]; therefore, loss of mitochondrial integrity seems to be a critical factor for the appearance of reactive astrocytes type A1 [62].

In addition, astroglial water channel aquaporin 4 (AQP4) seems to be involved in the control of inflammation, since AQP4-knockout mice demonstrate much more extensive gliosis in experimental neurodegeneration [63]. Molecules of AQP4 are specifically enriched in perivascular astroglial end-feet membranes (being co-localized there with dystrophins) to provide direct transport of water and though the BBB. Furthermore, AQP4 expressed at the astrocytic processes close to synapses (being co-localized there with potassium ion channel Kir4.1) may mediate interstitial fluid resorption, K+ clearance and extracellular space volume control upon neuronal activation in the NVU [64]. Particularly, AQP4 supports an exchange of cerebrospinal fluid (CSF) and interstitial fluid (ISF), and its activity should be taken into consideration while modeling neurodegeneration or other brain pathologies associated with abnormal CSF-ISF transport [65]. AQP4 takes part in the functioning of brain glymphatic transport, which is necessary for the elimination of numerous waste molecules during the sleep time; therefore, aberrant activity of astroglial AQP4 contributes to the impairment of glymphatic clearance mechanisms seen in dementia [66], or may increase the probability of neurodegeneration development after brain injury [67]. Thus, AQP4 becomes to be an attractive target for the therapy of brain edema and BBB breakdown [68,69].

Some authors suggest that aquaporins may function as a regulator of mitochondrial functions in other cell types [70], but whether it is applicable for brain cells remains to be examined. However, astroglial mitochondria inner membrane is equipped with AQP9, which is permeable for water and several organic molecules, particularly for lactate, presumably transferring this glycolytic metabolite into the mitochondria in conditions associated with OXPHOS alterations [71].

In experimental cerebral ischemia, astrocytes can act as donors of mitochondria for damaged neurons, and this mechanism is controlled by the activity of NAD+ glycohydrolase / CD38 and transfer complexes (EVs, tunneling nanotubes) in astrocytes [72]. Later, the similar mechanism was demonstrated in vitro in induced pluripotent stem cells (iPSCs)-derived neurons and astrocytes in the model of Alexander disease [73].

CD38/NAD+-glycohydrolase is expressed in many cell types as a multifunctional enzyme mainly acting on NAD+ to convert it to cyclic ADP-ribose with Ca2+-mobilizing activity [74,75,76]. Due to its oligomeric structure, CD38 behaves as a catalytically active transporter responsible for the generation and entry of cyclic ADP-ribose into the cell, whereas astroglial connexin 43 (Cx43) might be functionally coupled with CD38 to provide NAD+ access to the active site of CD38 [75]. Mitochondrial, cytosolic, and plasma membrane localization of CD38 makes it a candidate for a sensor of NAD+ levels in the cell [77]. In the brain, CD38 is expressed in neurons, astrocytes, and microglia, and is involved in the neurotransmitter action, neurodegeneration-induced neuroinflammation, tissue regeneration, and oxytocin release [78,79,80,81,82]. Some data suggest that CD38 is involved in aging-associated decline in intracellular NAD+ levels and corresponding progression of mitochondrial dysfunction in various tissues [83]. Astroglial cells elevate plasma membrane CD38 expression and activity as a response to excessive extracellular levels of glutamate or pro-inflammatory cytokines [84,85]. Thus, we may assume that CD38 overexpression in astrocytes could serve as a mechanism to rescue affected neighboring cells with severe mitochondrial dysfunction (i.e., neurons in excitotoxic conditions, or cells in the epicenter of inflammation) by intercellular mitochondria transfer. Therefore, even usually CD38 effects in glial cells are thought to be linked to the control of intracellular NAD+ pool, new data on the role of CD38 in mediating the cell-to-cell transfer of donor mitochondria, expanding opportunities of utilizing intercellular communication for diminishing the neurological dysfunction [86].

At the same time, hyperexpression of CD38 in astrocyes in neurodegeneration could be considered as a mechanism reflecting aging-related processes. It was previously confirmed for neuronal expression of CD38 as a causative factor of age-related NAD+ decline and mitochondrial dysfunction [87]. However, it is tempting to speculate that depletion of intracellular and intramitochondrial NAD + pool, due to high activity of CD38, might lead to the inhibition of a key glycolytic enzyme—NAD+-dependent glyceraldehyde-3-phosphate dehydrogenase—and suppression of glycolysis, which is a characteristic of brain tissue in Alzheimer’s disease [88].

Perivascular astroglial mitochondria may affect functioning of other NVU cells besides neurons. Particularly, mitochondrial enrichment detected in astroglial end-feet, forming close contacts with brain microvessel endothelial cells (BMECs) in brain capillaries [89] and rapid changes in mitochondria-controlled Ca2+ local concentrations in astrocytic processes suggest involvement of astroglial end-feet mitochondria in neurovascular coupling and regulation of microcirculation. Idiopathic intracranial hypertension manifested by a significant cognitive deficit is characterized by accumulation of pathological mitochondria with irregular shapes and swelling in perivascular astroglial end-feet [90], but whether the similar pattern of astrocyte mitochondrial dysfunction could be seen in Alzheimer’s type neurodegeneration remains to be unknown.

Lactate can maintain the functional activity of mitochondria, for example, in actively proliferating and energy-consuming cells, by increasing the intracellular pool of acetyl-CoA, induction of histone acetylation and gene expression. In addition to MCT-mediated transfer of lactate to neighboring NVU cells, we have previously shown that activation of lactate receptors GPR81 in BMECs stimulated mitochondrial biogenesis in vitro [91]. This finding suggests that astrocyte-endothelial metabolic coupling mediated by the production of lactate in astrocytes and its action on BMECs either via GPR81 receptors or due to MCT1-mediated uptake might be important for controlling BBB structural and functional integrity, and cerebral angiogenesis [1]. Based on in vitro BBB/NVU models, we proposed that there is a mechanism similar to the inverse Warburg effect: in areas of angiogenesis or BBB breakdown, endothelial cells stimulate perivascular astroglia to produce lactate, which acts at GPR81 receptors, thereby leading to the intensification of mitochondrial biogenesis and an increase in the proliferation of endothelial cells, and via reduction of MCT1-mediated influx of lactate into endothelial cells, thus contributing to angiogenesis and barriergenesis [1].

It should be noted that mitochondrial biogenesis stimulating effect is achieved by changing levels of intracellular NAD+ in BMECs. Recent findings show positive effects of NAD+ supplementation on prenatal cerebral angiogenesis and postnatal brain development [92]. A key activator of mitochondrial biogenesis PGC-1α is simultaneously an inducer of NAD + synthesis in various cells [93]. BMECs are sensitive to glycolysis suppression [94]; therefore, perivascular astroglia can maintain high mitochondrial activity of cerebral endothelial cells by permanent production and transport of lactate needed to restore OXPHOS followed by elevated NAD+ intracellular levels in BMECs. Impairment of astroglial glycolysis in Alzheimer’s disease brain [95] may result in inefficacy of this supportive activity of astrocytes and loss of BBB integrity.

One of the key stimulators of mitochondrial biogenesis is the *c-myc* oncogene, which activates the expression of genes that regulate the cycles of mitochondrial fusion and fission to expand the mitochondria content in the cell [96]. In actively proliferating cells, the expression of *c-myc* leads to the activation of the NAD+-synthesizing enzyme (NAMPT), an increase in the level of NAD+ and an increase in the activity of sirtuin 1 (NAD+-dependent histone deacetylase); thereby, mitotic activity and cell viability are preserved [97]. However, other evidence suggests that expression of *c-myc* causes an increase in glycolytic activity in proliferating cells and leads to a decrease in NAD + levels [98]. The uptake of lactate by cells stimulates *c-myc* activity in proliferating cells [99], but to what extent this may be true for postmitotic cells (mature neurons and astrocytes) within the NVU remains unclear. In endothelial cells, suppression of *c-myc* expression results in a pro-inflammatory senescent phenotype usual for vascular aging [100].

### 2.3. Mitochondrial Dynamics and Metabolic Control in BMECs and Pericytes

BMECs have unique expression profile, energy metabolism, high mitochondrial content, and secretory activity which changes upon stimulation of cerebral angiogenesis (developmental, activity-induced, or restorative) as well as in neurodegeneration [101,102]. BMECs within the NVU/BBB are characterized by specific properties distinct from endothelial cells in other tissues. Particularly, they have a very high density of mitochondria, low fenestration of the cell membrane, tight cell-to-cell coupling due to expression of numerous junction proteins, and specific pattern of transporters and receptors expressed in BMECs [101]. These features are important for the better control of BBB permeability to prevent entry of toxic or dangerous agents into the brain tissue, or to coordinate efflux of metabolites from the brain to peripheral blood.

It is well-established that mitochondria in endothelial cells (ECs) respond to various stimuli (oxygen concentration, hemodynamics) [103], and their activity depends on the availability of nutrients as energy substrates, efficacy of blood glucose, fatty acids and glutamine uptake, the cell status (quiescence or proliferation), as well as on changes in intracellular Ca2+ concentrations, functioning of other cell compartments and organelles (lysosomes, endoplasmic reticulum). As an example, evolution of ECs from the quiescent to proliferating/migratory phenotype (stalk and tip cells) is evident in angiogenesis. Such changes are associated with significant metabolic alterations: low glycolytic activity, NADPH regeneration in quiescent ECs are replaced with upregulated glycolysis, shifting glycolytic intermediates to biomass production, stimulation of pentose-phosphate pathway, excessive NAD+ regeneration due to high activity of OXPHOS, and fatty acid oxidation in mitochondria in angiogenic ECs [104]. The key glycolytic enzyme 6-phosphofructo-2-kinase/fructose-2,6-bisphosphatase 3 (PFKFB3) regulates angiogenesis by affecting the balance of stalk and tip cells: tip cells need extensive glycolysis for sprouting [105]. PFKFB3 and glycolysis are inhibited by laminar shear stress in ECs resulting in suppression of angiogenic sprouting [106], and it should be taken into consideration while assessing angiogenesis and barriergenesis in dynamic BBB in vitro models.

ECs consume a lot of glutamine for protein synthesis and replenishment of TCA, which is important for proliferating ECs: they show significant suppression of TCA when catabolism of glutamine is blocked [107]. It should be noted that excessive angiogenesis and establishment of porous BBB is a hallmark of Alzheimer’s disease brain [108], and glutamine-related issues might be of particular interest for understanding angiogenic activity of BMECs in neurodegeneration. Cerebral endothelial cells are able to convert glutamine into glutamate and to activate glutamate dehydrogenase to produce α-ketoglutarate, fueling TCA followed by elevated production of ATP (even under conditions of low glycolysis) in mitochondria that are highly expressed in BMECs [109]. This is in line with the observations of [110] on higher expression of excitatory amino acid transporters (EAAT) in adult BMECs compared with neonatal ones: at the earliest stages of brain development, lactate and ketone bodies are the preferable energy substrates whereas in the adult brain glucose is much more important in this context; therefore, adult BMECs should have additional mechanism to support TCA and to provide ATP synthesis in conditions of glucose shortage or deprivation. Another way to utilize glutamate in BMECs is to synthesize GABA with the activity of glutamate decarboxylase (GAD). Indeed, BMECs produce GABA, which modulates migration of embryonic neuronal cells [111], but whether this mechanism is present in the adult brain remains to be evaluated.

Architecture of cerebral microvessels is not constant in the adult brain since many physiological or pathological conditions are associated with enhanced angiogenesis, i.e., activity-driven angiogenesis coupled to neurogenesis [112], or damage-induced angiogenesis evident in the progression of Alzheimer’s disease [108]. Therefore, metabolic changes associated with the conversion of quiescent BMECs into angiogenic BMECs, or with the recruitment of endothelial progenitor cells (EPCs), are pivotal for successful completion of an angiogenesis program [101]. Thus, it is not surprising that impairment of mitochondrial function or dynamics is directly linked to the establishment of a functionally incompetent EC monolayer. For instance, defective mitochondrial respiration caused by deletion of Cr6-interacting factor 1 in BMECs leads to the reorganization of cytoskeleton, tight junction machinery, and loss of BBB integrity in mice in vivo [113].

BMECs are equipped to produce Aβ [114], expression and metabolism of APP in these cells is regulated by nitric oxide [115], and it might be relevant in the pathogenesis of cerebral amyloid angiopathy in Alzheimer’s disease. Aβ negatively affects mitochondrial activity in BMECs (suppression of OXPHOS and elevation of ROS production), and this effect is potentiated by high extracellular glucose levels resembling local insulin resistance in Alzheimer’s type neurodegeneration [116]. Disruption of Ca2+ homeostasis in BMECs caused by Aβ is accompanied by upregulation of OXPHOS (like in the inverse Warburg effect in neuronal cells described above) followed by overproduction of ROS, cell death, and development of cerebrovascular dysfunction [117,118]. Mitochondrial coenzyme Q10 pretreatment of ECs prevents Aβ accumulation in the cells, Ca2+ disturbances, ROS overproduction and mitochondrial dysfunction in the in vitro conditions [119]. We found positive effects of Q10 on cerebral angiogenesis and BBB integrity in hippocampus and amygdala in vivo [120] and in the BBB model in vitro [121].

Metabolic plasticity of BMECs is markedly changed in neuroinflammation. For instance, hyperactivation of Drp1 in BMECs exposed to LPS in the model of neuroinfection results in loss of tight junctions and elevated BBB permeability in vivo [122]. As we have shown before, LPS-induced neuroinflammation in the in vitro BBB model resulted in decreased expression of lactate receptors GPR81, lactate transporters MCT1, elevated lactate levels in the extracellular compartment, and reduced transendothelial electric resistance (TEER) of BMECs monolayer as a marker of the barrier integrity [123]. These data support the idea that astroglia-endothelial lactate-mediated metabolic coupling might be compromised in neuroinflammation.

Less is known about mitochondrial activity in brain pericytes. Activated pericytes in vessels of other tissues mainly depend on glycolysis than on mitochondrial respiration, and inhibition of key glycolytic enzyme PFKFB3 results in pericytes quiescence [124]. Oxidative damage of pericytes leads to fragmentation of their mitochondria and apoptosis [125]. Tight junction protein occludin expressed in brain pericytes affects glucose uptake and glycolytic activity in the AMPK-SIRT1-dependent manner, and takes part in the pericyte-astroglial metabolic communication within the BBB: in energy deprivation conditions, pericytes may share glucose with astrocytes or may even transfer mitochondria to damaged astroglia [126]. Moreover, there is an experimental approval that pericytes in mouse retina have functional tunneling nanotubes able to transfer mitochondria between the NVU/BBB cells to provide effective neurovascular coupling [127]. This mechanism might be affected in neurodegeneration, since in Alzheimer’s disease pericytes undergo extensive apoptosis, thereby contributing to BBB breakdown [10].

## 3. Application of In Vitro NVU/BBB Models for Studying Mitochondria-Related Changes in Cell Metabolism: Current Trends and Challenges

Metabolic plasticity of NVU/BBB cells driven by mitochondrial activity can be evaluated in vivo and in vitro; however, in vitro models provide more opportunities for dissecting signaling pathways and intercellular communication, mitochondria transfer or metabolites action in relation to NVU functional competence and BBB integrity. Up-to-date models are designed to reproduce the geometry of the NVU or BBB with endothelial cells and perivascular cells, and to examine the main aspects of the barrier activity [128,129,130].

Numerous in vitro BBB/NVU models are currently used to study the mechanisms of brain activity, BBB breakdown, to assess the permeability of candidate drugs and drug delivery system across the BBB, and to develop new methods for controlling BBB permeability in neurodegeneration. Existing BBB in vitro models are reconstructed from different types of cells (mainly from cerebral endothelium, pericytes, astroglia) of human or animal origin [131,132]. Up-to-date versions of the BBB models—static (transwell) and dynamic (microfluidic)—have significantly expanded research capabilities, but a number of issues remain unresolved, including the development of personalized NVU/BBB models that would allow studying barrier functions for a wide range of therapeutic agents for the benefit of particular patients.

**Figure 3:**
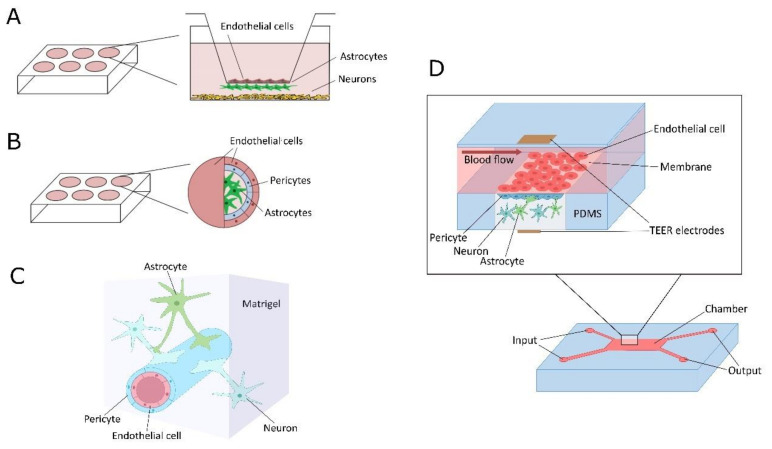
Blood–brain barrier in vitro models. (**A**)—2D static transwell model; (**B**)—3D static model; (**C**)—3D microdynamic/microfluidic/microvessel-on chip model; (**D**)—3D multicellular ensembles established from various components of the BBB. PDMS—Polydimethylsiloxane (polymeric organosilicon compounds); TEER—trans-endothelial electrical resistance.

Particularly, the following standard in vitro models are in use: (i) 2D static transwell models established with 2 or more cell types separated at the upper and lower compartments by the permeable membrane to provide development of a functional barrier; (ii) 3D static models made of cells submerged in hydrogels to support the complex geometry of the NVU; (iii) 3D microdynamic/microfluidic/microvessel-on chip models consisting of NVU/BBB cells grown in microchambers on appropriate membranes or synthesized analogues of extracellular matrix with permanent perfusion of extracellular fluid to mimic natural blood flow-induced changes in BMECs architecture and activity; (iv) 3D multicellular ensembles established from various components of the BBB to provide some artificial but functionally competent geometry, i.e., BBB spheroids, or iPCSc-derived cerebral organoids augmented with BMECs-derived “microvessels” [133,134,135,136,137,138]. Some of these models could be equipped with various sensors and devices for real-time imaging, assessment of transendothelial electric resistance, controlling metabolic parameters of cells, etc. (Figure 3).

A critical factor that determines the possibility to establish an adequate BBB in vitro model is the induction and achievement of properties of endothelial cells that are characteristic of brain microvessel endothelial cells (BMECs). Using BMECs but not ECs of other origin as a part of the models is more physiological to reproduce the main mechanisms of BBB development and functioning. As we have shown before, specific metabolic properties of BMECs makes them an interesting object for managing the barrier permeability in BBB models, or even in tissue models based on the cerebral endothelial monolayer (i.e., neurogenic niche in vitro model) [123,139,140,141,142,143,144].

For modeling neurodegeneration in vitro, the following approaches could be utilized in NVU/BBB models: (i) exposure of cultured cells to Aβ in vitro to reproduce amyloid-mediated acute cytotoxic effects; (ii) isolation of cells from the brain of transgenic mice with Alzheimer’s disease genotype for further co-culture and examination; (iii) isolation of cells from the brain of animals with non-genetic in vivo models of Alzheimer’s disease (i.e., intrahippocampal injection of Aβ): (iv) establishment of mixed models consisting of organotypic culture obtained from the animals with Alzheimer’s disease model and cells (i.e., BMECs) obtained from the intact animals; (v) application of genome editing or reprogramming technologies to get the in vitro model with the desired morphological and functional alterations resembling those in Alzheimer’s type neurodegeneration.

Adequate assessment of altered NVU/BBB structural and functional integrity as well as analysis of effects induced by drug candidates in the in vitro models is one of the challenges in neurobioengineering and neuropharmacology. Particularly, several approaches have been proposed for high-throughput screening (HTS) in vitro: (i) cell-based and biochemical assays that allow getting data on cell proliferation, differentiation, migration, cell-to-cell interactions, or signal transduction and metabolic status, respectively; (ii) application of omics technologies (genomics, transcriptomics, proteomics, metabolomics, etc.) even in the single-cell format if possible; (iii) application of real-time protocols and development of models with the integrated sensors for analyzing cell behavior and BBB permeability; iv) combining in vitro modeling with in silico modeling to provide informative and predictive protocols [145,146,147]. Thus, selection of a BBB in vitro model for HTS is mainly based on the parameters that should be taken for consideration: i.e., evaluating the wide spectrum of xenobiotics for their transport across the BBB might require simple 2D transwell systems, but cell-to-cell interactions and signal transduction pathways, i.e., mitochondria-driven mechanisms in BMECs required for barrier function, could be reproduced with high reliability in 3D models or in ones allowing for real-time analysis.

Currently, an important trend in BBB and NVU in vitro modeling is to obtain multicellular ensembles in the format of 3D- and 4D-models that reproduce the three-dimensional architecture of NVE and BBB, dynamic changes in the composition of the model during cell development, or metabolic control within the ensemble. For instance, spheroid NVU/BBB models are of considerable interest, as they consist of astrocytes and neurons surrounded by a layer of pericytes and BMECs, and have been shown to be useful in drug effects screening or neurotoxicological studies [148,149].

Another trend in reproducing the region-specific NVU or even personalized brain structures is to establish iPSCs-based BBB in vitro models or iPSCs-derived brain organoids (self-organized brain tissue) [150,151,152,153]. It was demonstrated that generation and co-culture of BMECs together with neurons or astrocytes derived from iPSCs improve the structural and functional integrity of the BBB as is confirmed by high TEER and an adequate expression pattern of tight junction machinery [152].

Organoids that have been developed in recent years (whole brain, integrating organoids, organoids on microfluidic chips, organoid transplants) have become quite actively used to study communication between different regions of the brain; for example, cortico-striatal pathways or intrahippocampal connections [154,155]. Moreover, application of protocols for recording organoid electrophysiological activity using multielectrode arrays (MEAs) identifies novel mechanisms that provide a tissue response to external stimuli [154]. Recently, brain organoids have been extensively discussed as new tools not only for use in neurobiology, neuropharmacology, and neurotoxicology in vitro, but also for solving problems of regenerative medicine [156]. However, a factor limiting the use of organoids is the gap in our knowledge about mechanisms of organoid development from iPSCs. Particularly, we do not know the details on a contribution of mitochondrial dysfunction to the disruption of developmental program in cells obtained from iPSCs, i.e., mutations of mitochondrial DNA abundantly present in iPSCs lead to significant functional defects in cells derived from iPSCs [157]. The same phenomenon was demonstrated in neurons derived from iPSCs obtained from persons with schizophrenia [158], but it remains unclear whether the presence of such defects is a direct consequence of mutations in the cell genome in a specific pathology, or whether they arise as a result of reprogramming and subsequent differentiation. However, recent studies report a more optimistic view on the preservation of mitochondrial structure and functions in iPSCs-derived cerebral organoids [159].

Another limitation in constructing 3D brain iPSCs-originated organoids links to reproduction of age-related mechanisms in the brain tissue resembling prenatal or early postnatal stages of development: several attempts have been performed to develop organoids for studying Alzheimer’s type neurodegeneration, but using the cells with prenatal transcriptional pattern and metabolism is still unresolved technical problem [160].

Since the iPSCs are predominantly glycolytic cells, the manifestation of mtDNA defects and the corresponding disturbances in mitochondrial respiration can be recorded in cells differentiated from iPSCs. Therefore, there is a need for detailed study of the expression patterns and preservation of mtDNA in iPSCs as well as of mechanisms that could eliminate cells with defective mitochondrial DNA. On the other hand, similar aspects have not been extensively studied in BBB models derived from iPSCs, even high mitochondrial activity in cerebral endothelial cells is a key factor in maintaining the integrity and selective BBB permeability, as we have discussed before. This dictates the need to study the metabolic activity of iPSCs-derived cells, which is important for validating BBB models suitable for use in neuropharmacology. In particular, a comparison of gene expression patterns in BMECs obtained from iPSCs and immortalized BMECs cell lines showed that BMECs differentiated from iPSCs have an enhanced expression of a number of genes that determine the proliferative potential of cells and their interactions with the extracellular matrix [161]. One may assume that underlying metabolic processes (balance of glycolysis and OXPHOS, or specific mitochondrial dynamics) could be affected as well. At the same time, it was clearly demonstrated that the use of iPSCs to obtain BMECs makes it possible to reproduce important specific signs of BBB damage in various types of brain pathologies, including neurodegeneration. Considering that at least 8 key protocols for obtaining BMECs from human iPSCs have been developed [162,163,164,165], this technology is becoming more and more in demand for the development of in vitro bioengineering products suitable for the market launch of BBB models. Particularly, as it was suggested, BBB in vitro models should match at least 12 main validation criteria related to the following issues: (i) structure (ultrastructure, wall shear stress, geometry); (ii) microenvironment (basement membrane and extracellular matrix); (iii) barrier function (transendothelial electrical resistance), barrier permeability, efflux transport); (iv) cell function (expression of BBB markers, turnover); (v) co-culture with other cell types (astrocytes and pericytes) [166]. However, getting more information on the status of mitochondria in iPSCs and iPSCs-derived cells might become a kind of integral criteria for matching the model requirements, and for further application of a model for HTS and studying brain diseases based on mitochondria-driven mechanisms.

Another challenge in the adaptation of 3D and 4D organoid NVU models to study neurodegeneration or to convert them into the reliable BBB models is the development of vascularized organoids; otherwise, metabolic plasticity and viability of cells within the organoids are compromised a priori. Lack of vascularization of organoids is a big problem for their application [167]. It is reasonable to assume that the use of cells that form the BBB, in particular, BMECs, as part of an organoid can overcome this limitation. Several approaches to vascularization of brain organoids have been proposed: induction of angiogenesis in organoid tissue, vascularization due to organoid transplantation into tissue in vivo, coverage of organoids with iPSCs-derived BMECs, application of bioengineered structures functioning as blood vessels [168,169,170,171], but intensive search for new vascularization protocols continues. Progress in this direction will give us new opportunities in the development of NVU in vitro models correctly reproducing energy metabolism and mitochondria-driven cell signaling.

## 4. Conclusions

In sum, mitochondria-driven NVU/BBB alterations have been extensively studied in various animal models of brain disorders, and there is growing evidence that modern approaches utilizing in vitro models are very prospective in the assessment of intercellular communications within the NVU/BBB. Development of new NVU-on-chip or BBB-on-chip as well as 3D NVU/BBB models and brain organoids suggests novel clues to understanding cell-to-cell interactions in the brain, which is highly required for translational studies, drug discovery, and establishment of novel analytical platforms.

**Figure 4:**
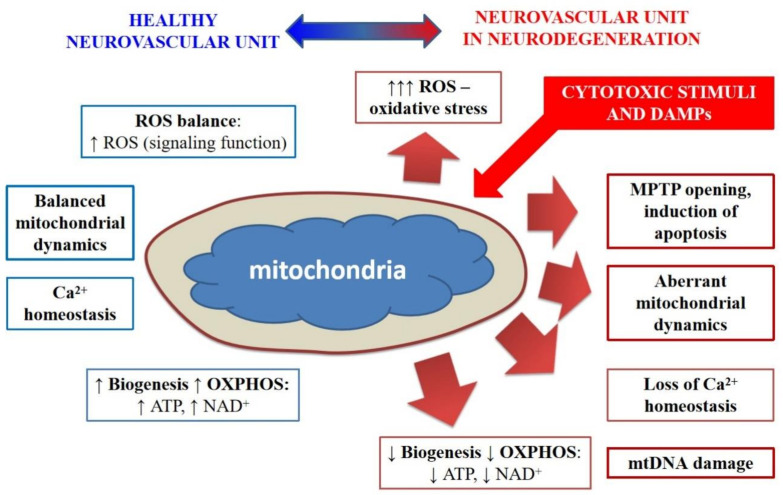
Mitochondria-driven pathways in the blood–brain barrier in healthy brain and neurodegeneration. Blue indicates physiological events, changes during neurodegeneration are indicated in red. ROS—reactive oxygen species; MPTP—mitochondrial permeability transition pore; mtDNA—mitochondrial DNA; OXPHOS—oxidative phosphorylation; NAD—nicotinamide adenine dinucleotide; ATP—adenosine triphosphate.

Experimental and clinical data on BBB and NVU functioning in (patho)physiological conditions, development of multidimensional and personalized in vitro models of NVU/BBB, and application of novel bioengineering solutions allow deciphering new molecular mechanisms of brain mitochondrial plasticity affected in neurodegeneration (Figure 4). At the same time, further progress in the establishment of in vitro brain tissue models is required for the development of novel drug candidates and drug delivery systems utilizing mitochondria as targets for efficient restoration of aberrant NVU activity, diminished BBB integrity, impaired cognitive reserve, and reduced regenerative potential in chronic neurodegeneration.
